# Application of a wide-field phantom eye for optical coherence tomography and reflectance imaging

**DOI:** 10.1080/09500340.2015.1045309

**Published:** 2015-06-23

**Authors:** Anthony Corcoran, Gonzalo Muyo, Jano van Hemert, Alistair Gorman, Andrew R. Harvey

**Affiliations:** ^a^ Physics and Astronomy Department, University of Glasgow, Glasgow, Scotland.; ^b^ Research Department, Optos PLC, Dunfermline, Scotland.

**Keywords:** phantom eye, optical coherence tomography, 3D printing, visual optics, image quality assessment, wide-field

## Abstract

Optical coherence tomography (OCT) and reflectance imaging are used in clinical practice to measure the thickness and transverse dimensions of retinal features. The recent trend towards increasing the field of view (FOV) of these devices has led to an increasing significance of the optical aberrations of both the human eye and the device. We report the design, manufacture and application of the first phantom eye that reproduces the off-axis optical characteristics of the human eye, and allows the performance assessment of wide-field ophthalmic devices. We base our design and manufacture on the wide-field schematic eye, [Navarro, R. *J. Opt. Soc. Am. A*, **1985,** *2*.] as an accurate proxy to the human eye and enable assessment of ophthalmic imaging performance for a $\pm 70^{\circ }$ external FOV. We used multi-material 3D-printed retinal targets to assess imaging performance of the following ophthalmic instruments: the Optos 200Tx, Heidelberg Spectralis, Zeiss FF4 fundus camera and Optos *OCT SLO* and use the phantom to provide an insight into some of the challenges of wide-field OCT.

## 1. Introduction

Eye phantoms are commonly used in the development and assessment of retinal imaging modalities ranging from adaptive optics [1] and oximetry [2] to established reflectance imaging techniques such as traditional fundus photography, scanning laser ophthalmoscopy (SLO) and recently optical coherence tomography (OCT) [3, 4]. We report a wide-field phantom eye (WPE) that provides a closer approximation to the optical properties of a real eye for wide-field imaging than do the planar-geometry phantom eyes that have been previously published for narrow-field imaging [5–7].

**Figure 1.**
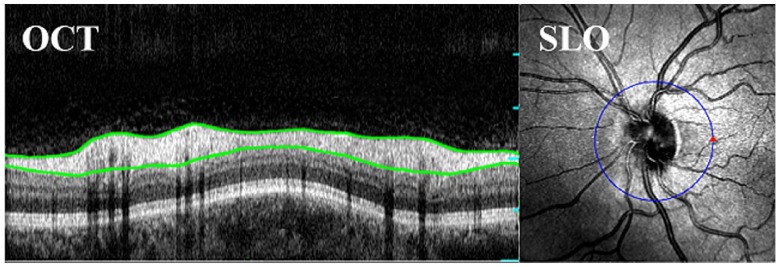
A Circular OCT B-Scan of a human retina with the retinal nerve fibre layer (RNFL) segmented in green, acquired on the Optos *OCT SLO*. (The colour version of this figure is included in the online version of the journal.)

The primary use of OCT, to image the fovea and optic disc, requires that a device can image a relatively modest $\pm 10^{\circ }$ external field angle. An example OCT B-scan around the optic disc is shown in Figure 1, including segmentation of the retinal nerve fibre layer (RNFL). The growing appreciation of peripheral pathologies has fuelled a trend towards increasing the field of view (FOV) of ophthalmic cameras and more recently OCT devices; achieving $\pm 80^{\circ }$ and $\pm 20^{\circ }$, respectively [8–10]. Current wide-field reflectance and fluorescence imaging modalities have been successfully used to monitor disease indicators in the retinal periphery such as non-perfusion and haemorrhaging in diabetic retinopathy and an increase in lipofuscin, which is associated with damage to the retinal pigment epithelium (RPE) during age-related macular degeneration [11–13]. The higher levels of optical aberrations and geometrical distortions at large field angles have been a major obstacle to quantitative assessment of these diseases in the retinal periphery, and furthermore the impact of these aberrations on retinal OCT scans has been difficult to quantify and compare between devices.

A standardised method for the assessment of accuracy, precision and performance comparison for OCT instruments has yet to be agreed upon [14–16]. Subjective comparison and assessment is hampered by the natural variations between the eyes from characteristics such as eye length, retinal morphology, severity of pathologies and the ability of a patient to fixate. We describe here the design, manufacture and application of a phantom eye for both OCT and reflectance imaging that mimics a comprehensive range of optical characteristics of the human eye, and has potential as a standard reference phantom for verification and calibration ophthalmic devices. Although many phantom eyes have been developed that enable the assessment of single metrics across a narrow FOV [4], to our knowledge our WPE is the first optical analogue of the eye to allow the assessment of both narrow and wide-field performance.

A modern ophthalmoscope is a complex system involving illumination and imaging optics, image enhancement, segmentation, montage and projection software, optical filtering and interface tools. Recent publications for narrow field eyes have prioritised the importance of retinal target design in the analysis of these capabilities. The optical design of a phantom provides an equally vital role of enabling both calibration and holistic characterisation of system performance. We attempt to achieve the following requirements for the WPE:

- High contrast features across a large field angle of the phantom retina to enable the measurement of imaging contrast and geometric distortion.
- Inclusion of physical features that are sufficiently small to enable determination of axial and transverse point spread functions (PSF) [6, 17].
- Calibrated axial structures that allow the assessment of performance metrics and image processing algorithms, such as those used for layer segmentation in OCT which is depicted in Figure 1 [5, 7, 18–21].
- Mimicking of retinal tissue e.g. for the validation of image enhancement algorithms [22, 23].

In Section 2, we present both the optical specifications and design considerations of the WPE. In Section 3, we outline the design and verification of the housing and interchangeable retinal targets. In Sections 4 and 5, we demonstrate the use of 3D printing to create two distinct calibration targets for OCT and SLO and discuss the effectiveness and limitations of this technology for retinal layer simulation.

## 2. Optical design of the WPE

We report a WPE that mimics the salient optical characteristics of a human eye across $\pm 70^{\circ }$ external FOV, equivalent to $\pm 90^{\circ }$ about the centre of the eye. The design of the WPE is based on the schematic eye model by Navarro et al., summarised in Table 1, since it closely replicates the optical aberrations and optical path lengths (OPL) of the human eye [25]. More complete schematic eye models have been reported, for example, containing GRIN lenses (mimicking the graded index property of the human eye) and reflecting variations in eye parameters with demographics [26]; however, their complexity makes manufacture impractical. The optical design was modelled and optimised in *Zemax*, replacing the biological material of the schematic eye with combinations of the standard glasses, BK7, FSi, CaF2 and PMMA; where the prescription of the glass and refractive surfaces were optimised to achieve a close similarity to the optical point spread function (PSF), aberrations, OPL and image distortion to the schematic eye.

**Table 1.** Optical prescription of the wide-field schematic eye for 532 nm, Navarro et al. [24] including the Zernike fringe description of the primary aberrations in units of wavelength.

Surface	Radius (mm)	Thickness (mm)	Ref. index	Conic
Corneal Lens	7.72	0.55	1.38	-0.26
Aqueous	6.5	3.05	1.34	0
Crystalline Lens	10.2	4	1.42	3.316
Vitreous	-6	16.3203	1.34	-1
Retina	-12	–	–	0
Aberration	Z4 (Defocus)	Z5 (Astigmatism)	Z8 (Coma)	Z11 (Spherical)
$\theta_{e}=3.5^{\circ }$	0.06	-0.01	0.03	0.00
$\theta_{e}=70^{\circ }$	-0.99	-5.71	0.45	0.06

Sufficient wide-field performance was achieved with a two lens system consisting of a fused silica cornea and ${\;\text{C}aF}_{2}$ crystalline lens. The WPE is filled with water to provide a close match to the chromatic dispersion of the ocular media of the human eye. The low refractive index of the lens materials provides a lower deviation to the OPL and distortion than other lens combinations. The use of ${\;\text{C}aF}_{2}$ also for the cornea would offer potentially superior OPL matching to the schematic eye; however, the poorer surface quality that is machinable for ${\;\text{C}aF}_{2}$ could cause excessive scattering of light at this interface. The optical performance has been optimised for wavelengths 532 and 830 nm to match the majority of illumination sources used in reflectance and OCT imaging. Finally, the prescription of the first lens was fixed to that of the schematic eye to maintain both a realistic corneal reflex and the effectiveness of reflex blocking mechanism in devices. The prescription for the wide-field phantom is summarised in Table 2.

**Table 2.** Optical prescription for wide-field phantom eye for 532 nm. Lens one and two are FSi and $\;{\text{CaF}}_{2}$ and are immersed in a water chamber. The primary Zernike fringe coefficients in units of wavelength, show a close similarity to those of the schematic eye.

Surface	Radius (mm)	Thickness (mm)	Ref. index	Conic
Lens One	7.72	0.55	1.46	-0.26
Water	6.5	3.05	1.33	0
Lens Two	11.22	3.93	1.44	0
Water	-5.9	16.32	1.33	-0.55
Target	-12	–	–	0
Aberration	Z4 (Defocus)	Z5 (Astigmatism)	Z8 (Coma)	Z11 (Spherical)
$\theta_{e}=3.5^{\circ }$	0.06	-0.01	0.03	0.00
$\theta_{e}=70^{\circ }$	-0.84	-5.25	0.41	0.08

**Figure 2.**
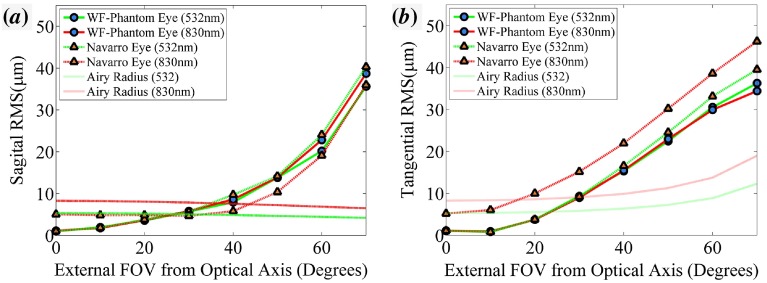
The optical performance of the WPE (circle) and Navarro eye (triangle). (*a*) The sagittal RMS radius for wavelengths 532 nm (green) and 830 nm (red). (*b*) The tangential RMS radius. (The colour version of this figure is included in the online version of the journal.)

**Figure 3.**
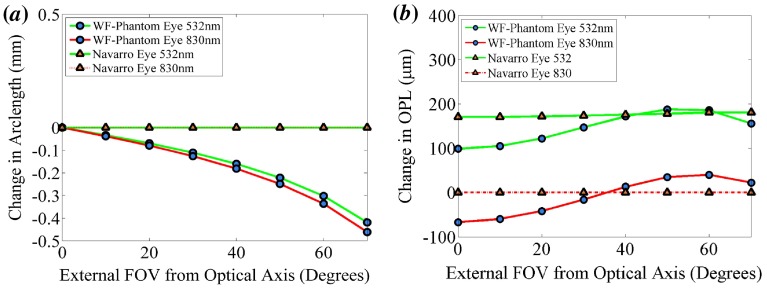
The geometric performance of the WPE (circle) and Navarro eye (triangle). (*a*) The 2D distortion of the WPE relative to the Navarro eye. (*b*) The difference in the OPL a ray must traverse from the cornea to the retina. (The colour version of this figure is included in the online version of the journal.)

A detailed description of the optical modelling of the WPE was previously published by us in conference proceedings [27] with the pertinent outcomes provided below. The RMS spot size and aberration coefficients at λ=532nm display a sufficient similarity to that of the schematic eye for all field angles, as shown in both Figure 2(*a*) and (*b*). The longitudinal chromatic aberration of the Navarro eye is greater than that of the WPE, indicating that further optimisation of the lens materials could improve the accuracy of the model. This chromatic defocus is unlikely to be observed in an OCT device incorporating automated focal correction. The maximum deviation in distortion between the WPE and Navarro eye, represented here as the arc length difference in the location of the chief ray intersection with the retina, is 0.47 mm at 830 nm as shown in Figure 3(*a*). This deviation would result in a relatively minor 2.5% reduction in FOV at 70${}^{\circ }$. The maximum change in OPL, shown in Figure 3(*b*) for 830 nm is 64 μm. This shift in OPL in a 2 mm A-scan constitutes a 3.2% deviation in the location of the reference arm; less than an order of magnitude lower than the standard deviation in axial eye length amongst people and hence, will not significantly impact automated algorithms commonly used for segmentation, dispersion correction or retinal flattening [28].

**Figure 4.**
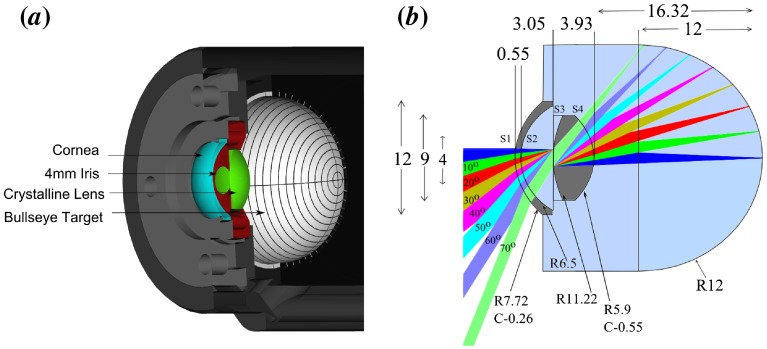
CAD drawings of all the components that comprise the phantom. (*a*) A cross section of WPE housing containing Target A. (*b*) A 2D-ray trace of the WPE. Each beam corresponds to a $10^{\circ }$ field angle. The surface apertures that must be maintained to ensure that there is no vignetting are (mm left to right) S1: 9.2 S2: 8 S3: 2 S4: 8. (The colour version of this figure is included in the online version of the journal.)

**Figure 5.**
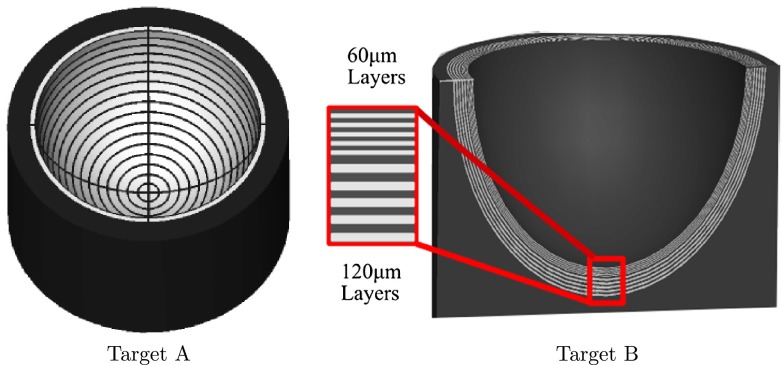
Left: Cross-sectional view the target with axial layers. Right: target containing a bullseye pattern. (The colour version of this figure is included in the online version of the journal.)

**Figure 6.**
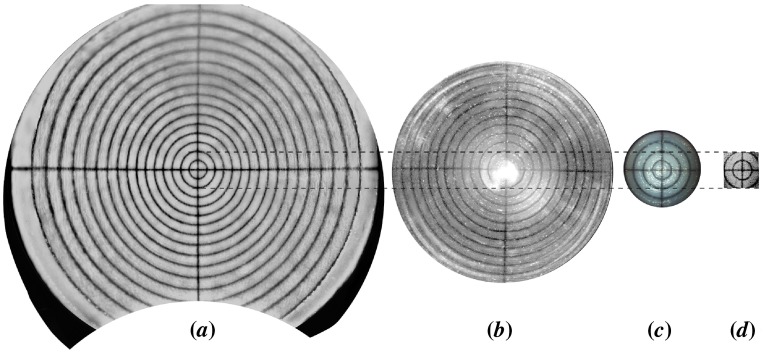
(*a*) Ultra-widefield SLO reflectance image shown using the Optos 200Tx with a stereographic projection. (*b*) Reflectance image from Heidelberg Spectralis with a wide-field non-contact lens designed for fluorescence imaging. (*c*) Zeiss FF4 fundus camera. (*d*) En face view of a C-scan of the Target A acquired using the optos *OCT SLO*. (The colour version of this figure is included in the online version of the journal.)

**Table 3.** FOV and distortion comparison of the five device modalities from the images shown in Figures 6 and 7. The change in magnification of the 200Tx and Spectralis use ring 12 as this is the furthest ring visible in both devices.

**FOV**	SLO		Fundus Camera	OCT	
	Optos 200Tx	Spectralis	Zeiss FF4	Optos (3D)	Optos (2D)
Arclength (mm)	41.8±0.1	28.1±0.1	9.0±0.05	4.9±0.05	6.8±0.05
Internal Field (${}^{\circ }$)	±99.9±0.5	±67.2±0.5	±21.5±0.2	±11.8±0.2	±16.2±0.02
External Field (${}^{\circ }$)	±83.3±0.4	±51.6±0.4	±15.2±0.2	±8.6±0.2	±12.0±0.02
ΔM(%)	+7.5±0.4	-3.5±0.4	-1.6±0.7	<1.0	<1.0

These modelled results indicate that the WPE design offers the closest manufacturable approximation to the optical and geometric properties of a real eye reported. Further improvement in the accuracy of this optical phantom relative to the human eye would require increased complexity in the schematic model. We believe that this optical design is sufficient for the assessment of performance and calibration of current ophthalmic devices. In the next section, we describe our progress with the use of 3D printing to create retinal-image targets and simulate tissue in the WPE.

## 3. Mechanical design and target fabrication

The critical dimensions for the phantom housing are shown in Figure 4. The lenses are mounted in a water filled anodised aluminium housing that has been machined with a 100μm precision.

Targets designed to be representative of the retina should accurately recreate both the structural composition and scattering profile of the retina. Three challenges exist for the fabrication of accurate layers. Firstly, conventional, high-resolution fabrication techniques such as laser ablation and guided deposition are ideal for planar surfaces, but their small depth of focus make their use cost prohibitive for large topographies. Secondly, fabrication techniques developed for the semiconductor industry such as of spin coating, used successfully across a narrow FOV by Baxi et al. [7], do not provide sufficient dimensional control for small transverse features. Thirdly, materials used in the phantom should provide comparable contrast to that found in the retina to be compatible with segmentation algorithms.

**Figure 7.**
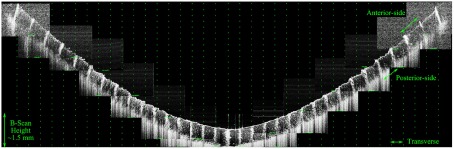
A montaged image of the entire hemisphere of Target A and displaying a 37.7 mm B-scan. The green dots have been provided to show the transverse distortion within the image. The number of pixels between each ring increases away off-axis. (The colour version of this figure is included in the online version of the journal.)

**Figure 8.**
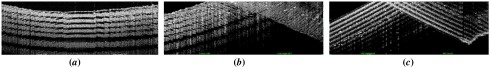
(*a*) On-axis image of Target B where all the layers are distinguishable (*b*) WPE rotated by $20^{\circ }$, equivalent to six rings in Target A shown in Figure 7, only 120μm layers remain distinguishable and a mirror artefact begins to obscure the image. (*c*) Off-axis cylindrical *scotch*-tape target with similar field angle used for (*b*). (The colour version of this figure is included in the online version of the journal.)

Recent advances in additive manufacturing often referred to as 3D printing, have made it possible to create arbitrary structures consisting of multiple materials with a tolerance of only a few microns. 3D printing has been demonstrated successfully in biophotonic imaging to calibrate field curvature in ex vivo tissue spectroscopy and to create microchannels for the calibration of hyperspectral reflectance imaging [20, 21]. The targets tested in the WPE were fabricated using the state-of-the-art *Objet Eden 350V* 3D printer which has 30μm transverse resolution, 16μm axial resolution in 3D printing and the ability to deposit multiple materials within a structure [29]. This 3D printing technique uses photo-polymerisation with sequential row deposition of multiple materials followed by UV curing to build each layer. The two target designs used to assess the suitability of 3D printing as a method for manufacturing OCT and reflectance imaging targets are shown in Figure 5.

Target A was designed to provide characterisation of the distortions and FOV of wide-field ophthalmoscopes. The design incorporates concentric rings of the high scattering and high-absorption materials *Verowhite* and *Veroblack* with an alternating width of 1 mm and 0.1 mm, respectively, intersected with a highly scattering cross hair. *Verowhite* contains titanium dioxide, commonly used as a scatterer in tissue simulation, [30] and *Veroblack* contains carbon black to increase absorption. The spacing between the ring transitions in the Target A were verified using calibrated imaging and the mean separation was found to be 1.09 mm ±10 μm. The images were acquired of Target A, both cleaved and sanded to half the lateral thickness and calibrated using a reference flat, which had been verified to ±10μm. In addition, possible distortion in the imaging system was mitigated by imaging a 2D grid. This separation is within 10μm of the specified value, with a precision below the image resolution of wide-field ophthalmic devices and therefore can be used to accurately measure distortion in an ophthalmoscope.

**Figure 9.**
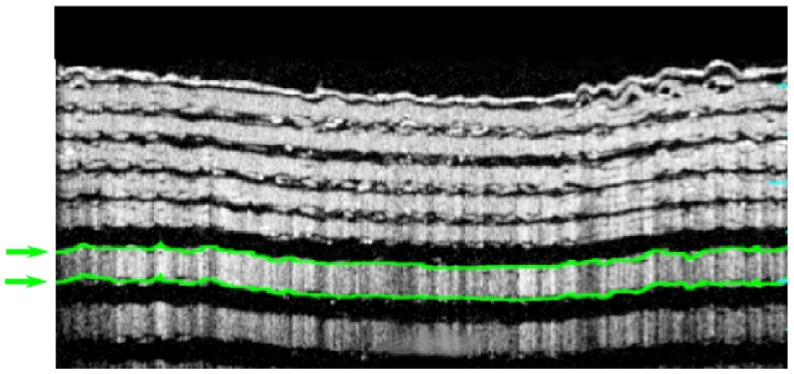
Left: Circular OCT B-scan of the Target B with a 10.7 mm circumference acquired using the method as Figure 1. The first 120μm has been segmented; shown in green. (The colour version of this figure is included in the online version of the journal.)

Target B was designed to investigate if 3D printing could be used to fabricate anatomical targets that allow the user to assess the accuracy and precision of OCT devices. The target incorporates five alternating thin layers of the highly scattering *Fullcure720* and *Verowhite* at 60μm thickness and five deeper alternating layers of 120μm thickness extending across the hemisphere of the target. *Fullcure720* is a transparent plastic, with similar refractive properties to PMMA (1.47) and is commonly employed for printing optical elements [31]. These thicknesses were found to be the lowest multiple of the 3D printing transverse resolution for which merging of layers did not occur at the off-axis locations. The relatively large 120μm thickness is nevertheless within the normal range of thicknesses for the RNFL [16] and so provides a pertinent scale size for assessment of OCT measurement performance. The layer thicknesses in Target B were verified using a research OCT system that was calibrated using the displacement of a submicron precision translation stage. Ninety B-scans were acquired with the top scattering and non-scattering layer segmented manually. From these images the mean layer thickness was measured as 59.9±2.8μm for each of the five thin layers and 121.3±3.2μm for each of the five thick layers, where the errors are given as the standard deviation of the measurements. OCT was used for the layer verification to eliminate distortions in the layer thickness that would occur from cleaving that target.

## 4. Performance assessment with Target A

Target A was used to measure the maximum FOV and the distortion of the images produced by five ophthalmic devices, as shown in Figure 6, following any internal distortion correction executed within the ophthalmoscopes. The results from analysis of these images are presented in Table 3. The distortion has been calculated as the change in image magnification, as given by Equation 1,

(1) $$\begin{array}{c} \Delta M=\left(N_{m}-N_{e}\right)/N_{e}, \end{array}$$

where $N_{m}$ is the number of pixels measured across the diagonal of the final ring imaged and $N_{e}$ is the expected arclength of the final ring, scaled by the number of pixels across the central ring.

**Figure 10.**
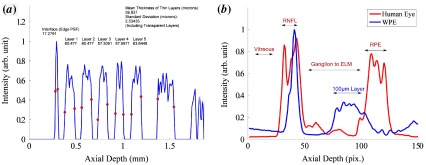
(*a*) Normalised intensity profile for the processed A-scan from Figure 8 (*a*). The red dots denote segmentation at the point of maximal intensity gradient. The intensity drops to zero for the transparent layers because the processed image has been thresholded to minimise noise. (*b*) The mean intensity across a B-Scan of both a human retina and a revision of Target B containing only 100μm layers, acquired from the spectrometer without image enhancement. (The colour version of this figure is included in the online version of the journal.)

**Figure 11.**
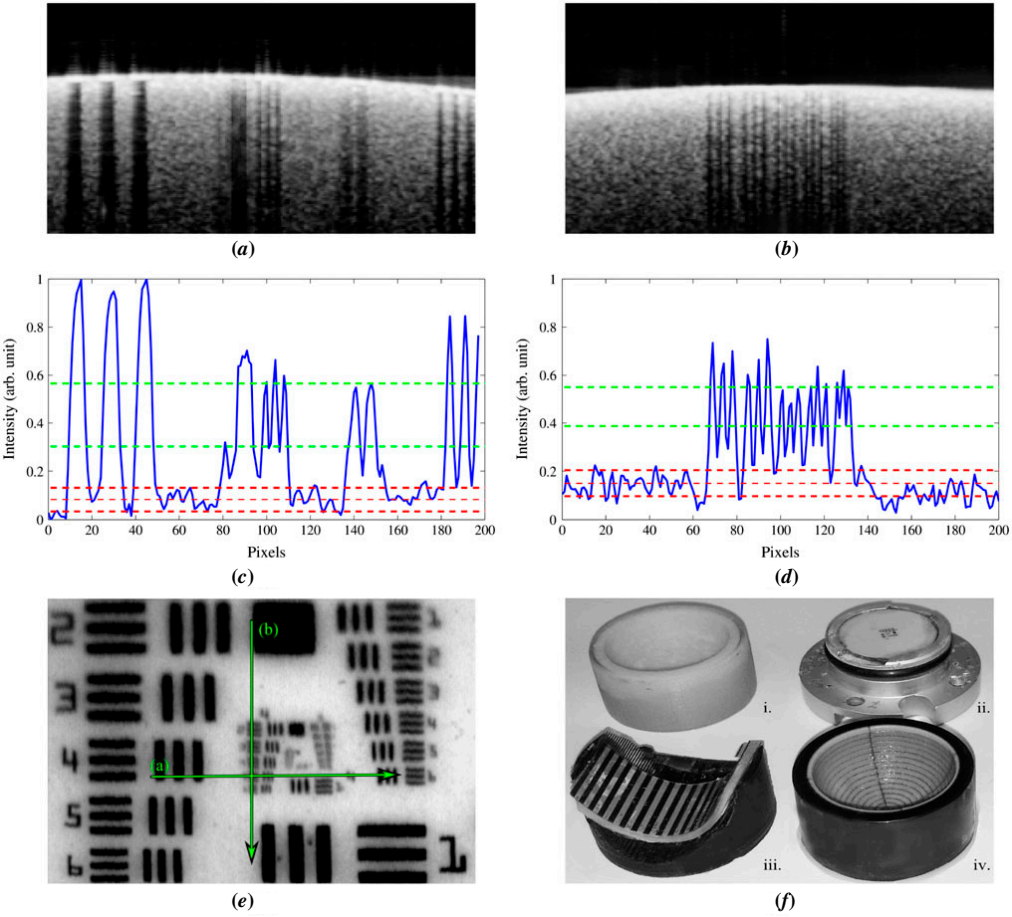
(*a*) Horizontal B-Scan through Group 4 Element 5 of the USAF Target (*b*) Vertical B-Scan through Group 4 of the USAF Target (*c*) Intensity profile of (*a*), averaged 50 pixels vertically and showing the minimum peak-to-valley contrast (green) exceeds the standard deviation of the noise (red) (*d*) Intensity profile of (*b*). (*e*) SLO image of the USAF Target with the lines in green (*f*) Image of the four targets used imaged (i.) Target B (ii.) USAF Planer Target (iii.) Bar chart cylindrical target (iv.) Target A. (The colour version of this figure is included in the online version of the journal.)

**Figure 12.**
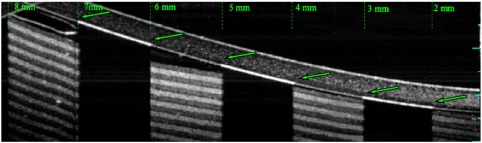
Commercial OCT image of the Ronchi ruling on the cylindrical Target with the edges used for LSF measurement highlighted in green. The feature width is 1 mm on a 0.25 mm thick film. (The colour version of this figure is included in the online version of the journal.)

The image shown in Figure 6(*a*) was acquired on the Optos 200Tx SLO (Optos Plc, Dunfermline, Scotland) [32] and is presented with a stereographic projection that preserves angles between features within the image. The maximum internal field angle was measured to be $\pm 99.9\;\pm \;0.5^{\circ }$, which is in agreement with the specified field angle by Optos of $200^{\circ }$ for the 200Tx. The change in image magnification of the 200Tx was measured at 7.5%, which can be attributed to the stereographic projection. This property must be considered during qualitative comparison of feature size between on-axis and peripheral features, such as use of the optic disc as a reference size. However, the known change in magnification from the use of a stereographic projection is easily calibrated in measurement tools.

The image shown in Figure 6(*b*) was acquired on a Heidelberg Spectralis SLO (Heidelberg Engineering, Vista, CA) with a non-contact wide-field lens. This lens is designed for fluorescence imaging and therefore Purkinje reflections and lens reflex are present at the centre of Figure 6(*b*). These artefacts do not contribute to the analysis of system performance. The maximum external field angle was measured to be $\pm 51.6\pm 0.4^{\circ }$, which is in agreement with the specified field angle by Heidelberg of $102^{\circ }$, when using the lens attachment. The change in image magnification of the Spectralis was measured at -3.5% and the device exhibits rotational symmetry. This minor barrel distortion is unlikely to influence clinical outcomes. The Zeiss FF4 fundus camera (Carl Zeiss Meditec, Dublin, CA) and the Optos *OCT SLO* using a raster C-scan shown in Figure 6(*c*) and (*d*) have significantly narrower FOV than the SLO devices from Optos and Heidelberg and as expected do not appear to exhibit significant distortions.

Target A was used to investigate the distortions in navigated OCT images of the human eye in which wide-field images are constructed from a montage of narrow-field images. The images were captured on an Optos *OCT SLO* that has an imaging depth of approximately 1.5 mm in water. The navigation of the WPE was achieved by rotation of the WPE by $10^{\circ }$ about the centre of the iris and manual adjustment of the reference arm OPL. A montage image of Target A with an internal FOV of a $180^{\circ }$ is shown in Figure 7. The OPL required to image the entirety of Target A was 5.4±0.1mm, which is in reasonable agreement with the 5.2 mm for the optical model in *Zemax*. The full external-FOV required to image the WPE was measured to be $\pm 70\;\pm \;2^{\circ }$, which is also in agreement with modelling. The consensus with modelling is critical for the use of the WPE to calibrate optical and geometric aberrations in wide-field images during automated scanning protocols.

Several properties of montaged OCT images produced from navigated imaging are evident from the B-scan image of the WPE in Figure 7: Firstly, the FOV of each component varies with projection angle; from $\pm 12.0^{\circ }$ for an on-axis image to $\pm 4.7^{\circ }$ for the furthest off-axis image. Secondly, the off-axis images display a reduced signal-to-noise ratio (SNR) as a result of the higher levels of optical aberration at wide-field angles, principally astigmatism and defocus; where the noise was measured as the standard deviation of the image intensity, excluding the target features. Finally, this form of navigation results in a change in magnification of 6.3±0.4% when considering only the transverse axis of the B-scan. However, this distortion is significantly higher when measuring along the arclength of the anterior side surface (25.5±0.4%) or the posterior side surface (20.9±0.4%). This variation between the anterior and posterior distortion across a modest 0.6 mm axial thickness indicates that the transverse measurement of the retinal periphery in OCT will require calibration for the axial eye length.

## 5. Performance assessment with Target B

Target-B was used to assess whether 3D printing could produce targets with sufficient quality to measure the accuracy and precision of an OCT device. OCT images of Target B are shown in Figure 8 for on-axis and off-axis imaging. On axis both the 120 μm and 60 μm layers are well resolved and exhibit high contrast layers; however, the low transverse resolution of the printing process leads to an irregular surface at larger field angles. This artefact causes the thinner layers to merge for larger field angles and also causes intensity streaking that is not present for a laterally continuous structure such as provided by stacked layers of adhesive clear and matt tape (polypropylene and cellulose adhered with acrylic) as shown in Figure 8(*c*).

These images suggest that current 3D printing technology does not yet have sufficient 3D resolution for the construction of wide-field retinas, nevertheless, on-axis, the images exhibit stable surfaces that can be used to compare and refine segmentation tools. The WPE was used to assess the accuracy of the Optos *OCT SLO* for measurement of the on-axis thickness of retinal layers within Target B as an analogue for segmentation of the RNFL, such as presented in Figure 1. The layer-thickness measurement tool on the device was used to segment the anterior side, 120μm scattering layer of Target B as indicated by the green lines in Figure 9.

Automated segmentation was performed on images obtained from 30 repeated B-scans of Target B mounted in the WPE. The mean in air thickness for this layer was measured as 130.1±5.6μm, 7% greater than the calibrated measurement of 121.3μm described in Section 3. The automated layer thickness was scaled to account for the ratio of the refractive index of *Fullcure720* (1.47) with that of the retina (1.36). The discontinuities in the 60 μm layers arising from the low-resolution printing prevented automated segmentation of this layer using the device software. From these images there was no observed change in the mean thickness of different layers; however, the variability in layer thickness does reduce with depth, as the radius of curvature of the layers is increased.

The axial PSF and the SNR are two additional performance metrics that are critical to the effectiveness of an OCT device. The axial PSF of the Optos *OCT SLO* device was measured from the resin/water discontinuity of Target B. The mean width (in tissue) of the reflection peak from this interface was measured as 18.7±2.6μm (full-width at half-maximum) sampled at 30 points across an image; an example intensity profile is shown in Figure 10. This measurement is consistent with the theoretical limit of 16.9μm expected for the 18 nm spectral bandwidth source used by the device and is lower than an equivalent measurement performed on a glass flat 21.7±3.1μm, acquired in air and scaled for tissue. The difference between measurements from Target B is possibly caused by the difference in dispersion between the measurements; however, this is unlikely to be significant because of the narrow-bandwidth of the source.

The propriety nature of the 3D-printed materials means that the optical properties (scattering, absorption and anisotropy) are not available from the manufacturer. To measure the similarity of the materials to tissue, the raw intensity B-scans from both Target B and a human eye, recorded with the research OCT device are shown in Figure 10(*b*), and shows a comparable overall intensity between the phantom and retinal tissue when linearly scaled, although the reflection from the vitreoretinal interface of the WPE exceeds that of the human eye as a result of the larger refractive index of *Fullcure720*.

## 6. Performance assessment with planar and cylindrical targets

Resolution patterns such as the USAF targets and ISO charts are commonly used to verify optical systems. The USAF-1951 resolution pattern was vacuum-sputtered in blue chrome onto a ceramic planar substrate with a precision of ±1μm. The Optos *OCT SLO* was used to acquire cross sections across both the horizontal and vertical axis of the USAF-Target as shown in Figure 11(*a*) and (*b*). Analysis of the intensity profile shown in Figure 11(*c*) and (*d*) demonstrated a fringe contrast of 28% for Group 4, Element 5 in the horizontal axis and 18% for Group 4, Element 6 in the vertical axis, measured using Equation 2

(2) $$\begin{array}{c} C=\frac{I_{peak}-I_{max}}{I_{peak}+I_{max}}>\sigma_{norm} \end{array}$$

These elements were the minimum feature size to exceed the noise of 4.1%, measured from the surrounding ceramic as the standard deviation of the normalised intensity. This corresponds to a resolution between 17.5μm-19.7μm, approximately double the pixel pitch, measured as 10.5μm. This measurement would be more representative of device performance during operation if the substrate had optical properties closer to those tissue [18].

The line spread function (LSF) is equivalent to the resolution performance of an imaging system, provided that the image being analysed has not been subject to non-linear image enhancement - such as thresholding or gamma correction. A Ronchi grating was printed on transparent Mylar polyester film (1.64), shown in Figure 11(*f*), and was attached to the cylindrical target, previously shown imaged in Figure 8(*c*). Flexible planar targets can be attached to a cylindrical shape to match the geometry of the eye, provided the imaging remains central along the axis perpendicular to the target curvature. The LSF of the Optos *OCT SLO* was measured from the navigated images that were linear in intensity, by taking the derivative of the transverse profile for each of the features highlighted in Figure 12.

The mean LSF was measured as 26.8±1.8μm, across an external FOV of $\pm 30^{\circ }$ with no significant variation recorded with respect to field angle. The larger value measured for the LSF in the cylindrical target than the resolution in the USAF target is most likely caused by the increased contrast of the USAF target features. The fixed resolution concurs with the modelling shown in Figure 2(*b*) that predicts that the WPE remains diffraction limited across this FOV. Measurement of the LSF for larger field angles was prevented by an increased noise obscuring the sampling of the feature transition. This limitation could be overcome by increasing the contrast of the bars or averaging pixels vertically along a substrate with similar scattering properties to tissue. The measurement of the LSF for device verification will be important in the internal comparison and calibration of wide-field and navigated OCT devices; however, it is limited accuracy for commercial comparison where the user is unlikely to have full control over the parameters of image enhancement.

## 7. Conclusion

The WPE enables the assessment of OCT, SLO and fundus camera performance across a significantly wider FOV that has previously been reported. The optical properties of the WPE matches those of the Navarro schematic eye in terms of PSF, aberrations, OPL and distortion for field angles less than $\pm 70^{\circ }$, although with slightly lower chromatic aberration. The wide-field images recorded with OCT and SLO show that this variation does not degrade the characterisation of the imaging performance in the retinal periphery.

The flexibility the WPE allows customised retinal targets to be used to characterise specific modalities and metrics. Two 3D-printed targets have been fabricated for the WPE with features across $\pm 90^{\circ }$. The large-scale features, such as the bullseye rings and hemispherical shape of the vitreoretinal interface, have been used to measure the FOV, variation in optical path length, distortion and axial-PSF with field angle. The materials used for 3D printing have been shown to have scattering properties sufficiently similar to that of retinal tissue to generate OCT images for the assessment of OCT-device segmentation algorithms and SNR performance. The optical properties for commonly used ophthalmic wavelengths should be measured in future to allow for an improved replication of tissue characteristics. The transverse precision of 3D printing is currently insufficient for the assessment of resolution across a wide-FOV.

Two planar targets were created using conventional imaging patterns, these were used to provide resolution assessment both on-axis and across an external field of $\pm 30^{\circ }$. Improvements in 3D printing technology or hybridising of the targets with other high-resolution fabrication techniques offer the potential to replicate the anatomy of the eye on low-cost, customisable retinal targets, which combine both geometric accuracy with sub-resolution features. The inclusion of fluorescing particles or biologically realistic vasculature will allow additional ophthalmic imaging modalities to be addressed by the versatility of 3D printing.
